# Greyhound racing ideal trajectory path generation for straight to bend based on jerk rate minimization

**DOI:** 10.1038/s41598-020-63678-1

**Published:** 2020-04-27

**Authors:** Md. Imam Hossain, David Eager, Paul D. Walker

**Affiliations:** Faculty of Engineering and Information Technology, University of Technology Sydney, Broadway, 2007 NSW Australia

**Keywords:** Mechanical engineering, Applied mathematics, Civil engineering

## Abstract

This paper presents methods for modelling and designing an ideal path trajectory between straight and bend track path segments for racing greyhounds. To do this, we numerically generate clothoid and algebraic curve segments for racing quadrupeds using a sequential vector transformation method as well as using a helper equation for approaching ideal clothoid segments that would respect greyhound kinematic parameters and boundary conditions of the track. Further, we look into the limitations of using a clothoid curve for racing dog track path design and propose a smooth composite curve for track transition design which roughly maintains G3 curvature continuity for smooth jerk to overcome limitations of a clothoid transition. Finally, we show results from race data modelling and past injury data, which provide a strong indication of clothoid curve segments improving the dynamics and safety of racing greyhounds while reducing injuries.

## Introduction

In the greyhound racing sports industry, injuries to dogs are highly prevalent^1^. The sport has grown exponentially in recent years due to live wagering accessibility and various revenue sharing programs^2^. As a result, it has become evident that better track design is required to reduce the likelihood of racing greyhound injuries at the tracks. Observations^3^ confirmed that in greyhound racing congestion occurs at the entrance to the first bend. Also, researchers theorized that a smooth-running path is required for curved track design without which quadrupeds are more likely to lose coordination at specific transitions^4^. Similarly, it was shown that various track shapes have considerable effects on greyhound injury rates indicating track curvature influences^5^. When it comes to track shapes and smooth paths, transition curves are an essential part of path design in many areas such as road design and train track designs^6^. Similarly, transition curves help reduce disturbances in quadruped gait symmetry^4^. This is because, quadrupeds are subject to a centrifugal force which induces an outward pull on the curved track path, forcing quadrupeds to deviate from navigating the track path^4^. Theoretically, a transition curve would also assist navigation of the body around the curved path even if it is not sufficiently banked^7^.

Clothoid transition curves are extensively found in road and rail track designs such as it was found from the analysis that the Tokaido Shinkansen high-speed rail uses a 600 m clothoid transition in one of the 2.1 km radius bends to achieve a maximum travelling speed of 270 km/hr with minimal track path camber. Clothoid curves are essential for generating continuous curvature paths with straight and perfect arc segments^8^. This is achieved by linking constant curvature segments with clothoid segments^8^. For example, a clothoid can join a line and a circle with G2 curvature continuity where both the tangent vector and curvature at the line-circle intersection are continuous^9^.

The performance of clothoid and other transition curves trajectories can be effectively analyzed by looking into their curvature profiles. Curvature is an import factor in trajectory designs as it affects the maximum speed a vehicle can travel without skidding or whether the pilot of an aeroplane suffers blackout as a result of g-forces^10^. Also, a valid curve is one which respects upper bound curvature constraints set by kinematics properties of moving bodies^11^.

In this paper, we illustrate numerical methods to approach clothoid curves and other transition curves to model and generate smooth running paths for greyhound racing. We also show galloping greyhound trajectory performance, relating it to injury rates and track shapes. The paper is organized as follows. Sections one and two describe greyhound trajectory and trajectory dynamics, respectively. In sections three and four, clothoid transition generation and approaching ideal clothoid transitions for racing greyhounds are presented. Ideal transition curves developed for galloping greyhounds are presented in section five. Finally, section six evaluates racing greyhound trajectory performance for existing tracks.

### Trajectory of a racing greyhound

In the greyhound racing industry, the trajectory of a racing greyhound is oftentimes overlooked for track designs and injury prevention measures despite its significance in dynamic outcomes for the animal. One key parameter which determines the trajectory of a racing greyhound is the curvature of its running path. Curvature, ***κ(s)***, is the change of heading relative to distance travelled^8^. Also, the curvature can be thought of as the inverse of the radius of curvature, which denotes the turning radius at any point in the path^12^. Furthermore, a related variable, sharpness ***α***, is the change of curvature for distance travelled which also forms the basis for constructing continuous curvature path trajectories^8^. While designing a path, curvature change must remain smooth throughout the trajectory of a moving object as the centrifugal acceleration experienced is directly proportional to the path curvature^12^. As a result, in trajectory generation for motion planning the smoothness of a trajectory is directly related to the smoothness of its curvature profile^13^. Likewise, for the path to be feasible, it must conform to continuous position, heading, as well as curvature at all points^8^. Now, if the path of the trajectory is defined by a function ***y*** = ***f(x)*** then the radius of curvature ρ at any given point can be found from the following equation:^14^

1 $$\rho =\frac{{\left[1,+,{\left(\frac{dy}{dx}\right)}^{2}\right]}^{3/2}}{|\frac{{d}^{2}y}{d{x}^{2}}|}$$

Then, the curvature is,

2 $$\kappa =\frac{1}{\rho }$$

**Figure 1:**
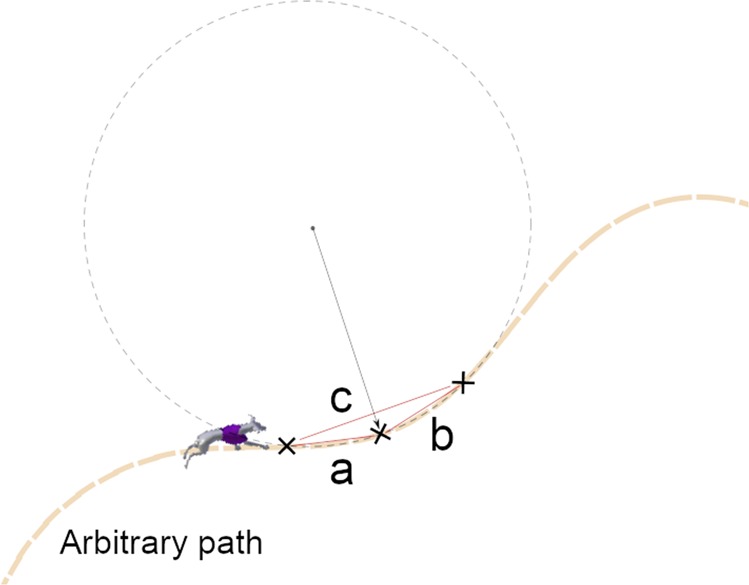
Calculating an arbitrary path’s instantaneous radius of curvature using data points lying on the path.

However, if the path of the trajectory cannot be translated into a continuous function, then any three adjacent data points lying on the path can be used to calculate the radius of curvature at any given point using the circumradius of a triangle formula^15^. The circumradius formula (3) provides the radius of the circumcircle of a triangle which is inherently cyclic^16,17^. The triangle is defined by the adjacent data points lying on the path, as shown below in Fig. 1.

3 $$\rho =R=\frac{abc}{4A}$$

Where, ***a***, ***b***, and ***c*** denote the three sides of a triangle defined by three adjacent data points on the path, and ***A*** is the area of the triangle.

An ideal racing greyhound trajectory would involve looking into two major control factors, greyhound heading which deals with curvature and sharpness of the running path and greyhound kinetics which deals with the acceleration/deceleration of a greyhound.

### Racing greyhound trajectory dynamics

The trajectory of a racing greyhound induces dynamic greyhound conditions such as centrifugal acceleration, centrifugal jerk, and greyhound heading yaw rate. It also influences racing greyhound states such as leaning, braking forces as a result of ground reaction force, centripetal force, stride frequency, and stride length. A sharp discontinuity in any of the dynamic conditions would result in a significantly unpredictable dynamic imbalance for a racing greyhound. During racing, such a situation would put a greyhound in considerably uncontrollable situations where there are already racing situations such as congestion and tight bends with variable track cross-falls along the width of the tracks. To design a trajectory for racing greyhounds which would meet the specific track design goals, the trajectory performance can be evaluated by looking into two dynamic factors of racing greyhounds namely: centrifugal jerk and yaw rate. These two factors are highly sensitive to the trajectory performance of an object in motion as both are related to the radius of curvature of the trajectory.

### Modelling centrifugal jerk

Jerk is the rate change of acceleration. Like centrifugal acceleration, the effect of jerk is also experienced in the body^18^. Essentially, jerk is the increasing or decreasing of the force in the body^18^. Eager, *et al*.^18^ explains the use of jerk as a measure of safety in various disciplines including mechanical engineering and civil engineering as well as its application in greyhound racing. Lower jerk values are essential as they indicate that the change in centrifugal acceleration is minimal for a greyhound while it is navigating its trajectory. For, humans, there are derived maximum acceleration change and corresponding time duration for this change for roller coaster rides^18^. No such derivations exist for racing quadrupeds yet. As a result, modelling of the centrifugal jerk for racing animals becomes an essential part of optimum trajectory generation.

The first step to centrifugal jerk analysis is finding the instantaneous radius of the trajectory or calculating the radius of curvature at all points in the trajectory path. For cars and trains, calculation of the instantaneous radius of curvature is found by using geometric primitives and splines and approximated using continuous functions as their respective heading change is continuous. However, for greyhounds, the heading change by the greyhound is not continuous and expected to occur at every stride. Furthermore, greyhounds are known to have a stride frequency greater than 3 Hz^19^. This implies a greyhound would change its heading if required more than three times a second where the magnitude of each heading change could vary from stride to stride. Therefore, we can gather all the location coordinate data for strides of a single racing greyhound and calculate the instantaneous radius of curvature ***ρ*** of the racing greyhound using either the circumradius formula (3) or the perpendicular bisectors method. Then, we can calculate the racing greyhound’s instantaneous centrifugal acceleration from the instantaneous speed and radius of curvature. Finally, the instantaneous jerk is derived from the rate of change in the centrifugal acceleration.

### Modelling yaw rate

The yaw rate is the rate change of heading or turning. It relates a racing greyhound’s angular displacement to its forward speed. It also provides an indication of the stability of the path a racing greyhound is taking. For example, it was shown from the race kinematic simulation and race data that racing greyhounds’ yaw rate is not smooth immediately after jumping out from the starting boxes^20^. For a constant radius curve path, the yaw rate is simply the radius of curvature over speed (4) which is used for calculating a vehicle’s momentary radius of turn. For a racing greyhound trajectory, the yaw rate can be directly related to the sum of the lateral forces. A lower yaw rate would indicate lower lateral forces such as centrifugal force and frictional force acting on a greyhound. To maintain a smooth trajectory, a racing greyhound needs to maintain a smooth yaw rate. However, since the speed of the racing greyhound varies over time as well as the lateral frictional forces from the traction ground, maintaining a smooth yaw rate would also require careful balancing of these two factors while designing tracks to facilitate a smooth trajectory for a racing greyhound.

4 $$\dot{\psi }=\frac{s}{\rho }$$

### Clothoid track segments for deriving natural racing greyhound trajectory

The clothoid segment is a curve known for its curvature being proportional to its length^21^. This property of the clothoid is useful as it allows the gradual development of centrifugal acceleration or can act as centrifugal acceleration easement, which significantly reduces the risk of accidents occurring^12^. Recent research shows that there are different types of curves already developed, which can be used as centrifugal acceleration easement curves^12^. For example, Quintic polynomial and B-splines functions are computationally less expensive and also able to provide curvature continuity for curve design^13^. However, the drawbacks of these functions are complex curvature profiles which are hard to follow as they are not necessarily smooth^13^. This is where clothoids are useful as their curvature profile is a straight line making them easy to follow^13^. Furthermore, clothoids are characterized by a linear curvature, allow minimizing of curvature variation where piecewise clothoids exhibit excellent smoothness properties^22^. For these fundamental reasons, currently clothoids are extensively found in road design and robot path planning to achieve smooth transitions in the trajectories^22^.

We found that clothoids are essential at the race track not only for developing smooth path trajectories but also for reducing the likelihood of certain types of race dynamics hazards. From the race videos, it was noted that a greyhound is more likely to change lanes to a higher radius upon entering the first bend. This could be due to the track bend lacking adequate transition to accommodate for greyhound natural instantaneous yaw rate change and leaning rate change limits. As a result, the prospect of the greyhound bumping into another nearby greyhound increases significantly. This specific race dynamic outcome can be reduced or nearly eliminated if the track path has clothoid segments which match natural greyhound heading turning rate change limits.

### Generating clothoid segments for track path design

There are many methods available for computing the clothoid. Most methods involve approximations to the clothoid^21^. For example, it can be approximated by high degree polynomial curves^23^, such as by an S-power series^24^ as well as by an arc spline^9^. Also, continued fractions and rational functions are commonly used for approximations^9^. A more recent development in the spline primitives found in much computer-aided design software makes it easy to approximate a clothoid while respecting boundary conditions such as curvature and tangent continuity. Also, spline primitives are known for good and fast controllability with positional and tangential constraints making them ideal for various applications^22^. Each of the methods available results in different degrees of accuracy and may not be suitable for efficient greyhound track path design purposes. This is mainly due to less controllability in generating a clothoid according to greyhound kinematics. Moreover, to accommodate the clothoid segment into the path design, a coordinate respecting system must be incorporated or derived from the existing clothoid methods which respects different design boundary conditions.

### Computing clothoid curves using existing methods

The most common method of computing a clothoid can be found in its definition in terms of Fresnel integrals^24^ where it is computed using the Fresnel sine and cosine functions as shown in Eqs. (5a) and (5b) and using some forms of Taylor series expansions on the functions which converge for an independent variable^8^. Series expansion functions are extensively used because the clothoid defining formulas are transcendental functions^21^. The parametric plot of Fresnel sine and cosine functions provides coordinate values of the clothoid curve. However, this does not respect any form of unit scaling or boundary conditions as well as not allowing computing the clothoid for a specific rate of change of curvature, sharpness or smoothing applications. Similarly, Eqs. (6a) and (6b) give an approximation of Fresnel sine and cosine functions which converge for all independent variables ***x***. Another common method involves utilizing auxiliary functions^8^, as shown in Eqs. (7a) and (7b).

5a $$S(x)={\int }_{0}^{x}\sin ({t}^{2})dt$$

5b $$C(x)={\int }_{0}^{x}\cos ({t}^{2})dt$$

6a $$S(x)={\int }_{0}^{x}\sin ({t}^{2})dt=\mathop{\sum }\limits_{n=0}^{\infty }{(-1)}^{n}\frac{{x}^{4n+3}}{(2n+1)!(4n+3)}$$

6b $$C(x)={\int }_{0}^{x}\cos ({t}^{2})dt=\mathop{\sum }\limits_{n=0}^{\infty }{(-1)}^{n}\frac{{x}^{4n+1}}{(2n)!(4n+1)}$$

Equations (5a) and (5b) then can be written in the auxiliary function form, as shown below:^8^

7a $$C(x)=\frac{1}{2}+f(x){\rm{s}}{\rm{i}}{\rm{n}}(\frac{\pi }{2}{x}^{2})-g(x){\rm{c}}{\rm{o}}{\rm{s}}(\frac{\pi }{2}{x}^{2})$$

7b $$S(x)=\frac{1}{2}-f(x){\rm{c}}{\rm{o}}{\rm{s}}(\frac{\pi }{2}{x}^{2})-g(x){\rm{s}}{\rm{i}}{\rm{n}}(\frac{\pi }{2}{x}^{2})$$

Where auxiliary functions ***f*** and ***g*** are defined as:

8a $$f(x)=\left(\frac{1}{2}-S(x)\right){\rm{c}}{\rm{o}}{\rm{s}}(\frac{\pi }{2}{x}^{2})-\left(\frac{1}{2}-C(x)\right){\rm{s}}{\rm{i}}{\rm{n}}(\frac{\pi }{2}{x}^{2})$$

8b $$g(x)=\left(\frac{1}{2}-C(x)\right){\rm{c}}{\rm{o}}{\rm{s}}(\frac{\pi }{2}{x}^{2})+\left(\frac{1}{2}-S(x)\right){\rm{s}}{\rm{i}}n(\frac{{\rm{\pi }}}{2}{x}^{2})$$

Likewise, for auxiliary function definition of the clothoid a good rational approximation to compute the clothoid is using the following auxiliary functions^8^.

9a $$f(x)=\frac{1+0.926x}{2+1.792x+3.104{x}^{2}}$$

9b $$g(x)=\frac{1}{2+4.142x+3.492{x}^{2}+6.670{x}^{3}}$$

Moreover, recently, researchers developed more efficient numerical methods where one such method is using arc length parameterisation^12^. While analytical methods lack parameterisation for different application case scenarios researchers are becoming more reliant on developing numerical techniques for computing the clothoids.

### A numerical approach for generating the clothoid curve transitions for racing greyhounds and other quadrupeds

**Figure 2:**
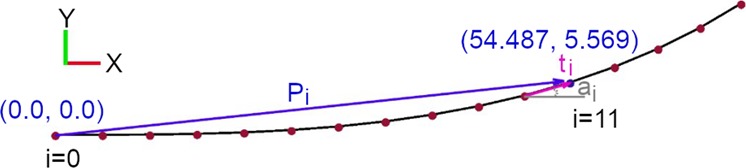
Racing greyhound clothoid path generation using numerical method parameterization.

**Figure 3:**
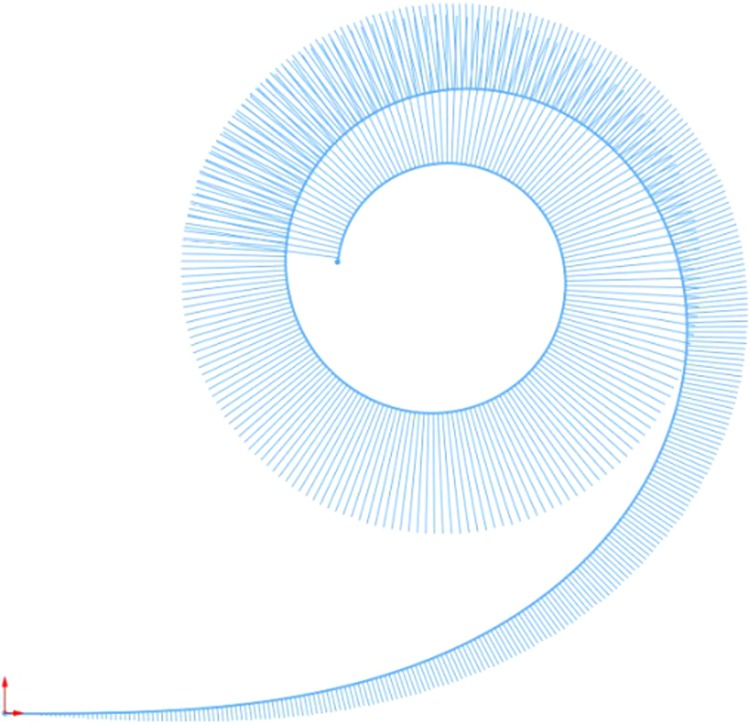
A clothoid curve with curvature combs containing 250 single meter segments and with a turning acceleration of 0.02 degrees per segment.

It is evident that existing methods lack greyhound kinematic parameterisation for racing greyhound transition design purposes. A numerical method is generally preferred as a first approach for incorporating different parametrisation into the clothoid curves. To develop a numerical technique for the clothoid which incorporates greyhound kinematics variables, we looked into the characteristics of the mathematical model of the clothoid curve. A clothoid curve transition accomplishes a gradual transition from the straight to the circular curve of the constant radius where the curvature changes from zero to a finite value. As a result, the tangent vector ***t***_***i***_, which lies on the clothoid curve, also gradually rotates from zero to a finite angle Fig. 2. Furthermore, let us assume a greyhound changes its heading with every stride as noted from the race data and galloping gait of a greyhound. With these two crucial pieces of information relating to the clothoid curve tangent vector and the greyhound heading step-change length, we can apply vector transformation to generate a clothoid curve positional vector ***P***_***i***_ Fig. 2. Now, we define the clothoid tangent vector as a function of greyhound stride length constant as denoted by transition segment length and a variable denoted by transition deflection angle. The transition deflection angle ***a***_***i***_ defines the local rotation of the clothoid curve tangent vector at a specific transition segment location ***i*** relative to the horizontal axis. Moreover, as a clothoid curve transition would gradually increase its curvature with constant curvature acceleration, the transition deflection angle ***a***_***i***_ is a function of the transition deflection angle acceleration constant. The transition deflection angle acceleration *d* defines the rate change of curvature per transition segment length of the clothoid curve, which essentially tells us how quickly the clothoid tangent vector rotation is accelerating. Finally, once the transition deflection angle is calculated for local ***i***th transition segment, the clothoid curve positional vector can be calculated as shown in Fig. 2 and Eq. (11). To generate the entire clothoid curve for the specified number of transition segments by the constant ***n*** the process of translating and then rotating the clothoid tangent vector is iterated to get the clothoid positional vectors for all the transition segments. For example, Fig. 3 shows a clothoid curve generated using this method when transition segment length ***s*** equals 1 m, the number of transition segments ***n*** equals 250 and transition deflection angle acceleration ***d*** is 0.02 degrees.

***d*** = transition deflection angle acceleration

***a***_***i***_ = transition deflection angle relative to horizontal axis

***s*** = transition segment length

***n*** = number of transition segments

***t***_***i***_ = transition tangent vector

***i*** = transition segment number

10 $${t}_{i}=f(s,{a}_{i})=[\begin{array}{c}cos({a}_{i})\times s\\ sin({a}_{i})\times s\end{array}]$$

11 $${P}_{i}=f({t}_{i},{P}_{i-1})={P}_{i-1}+{t}_{i}$$

Where,

$${a}_{i}=\mathop{\sum }\limits_{k=1}^{i}d\times i=1\times d+2\times d+3\times d+\ldots +i\times d$$

And,

$$d\times i\propto \kappa $$

Where ***κ*** denotes the curvature of the clothoid curve.

**Figure 4:**
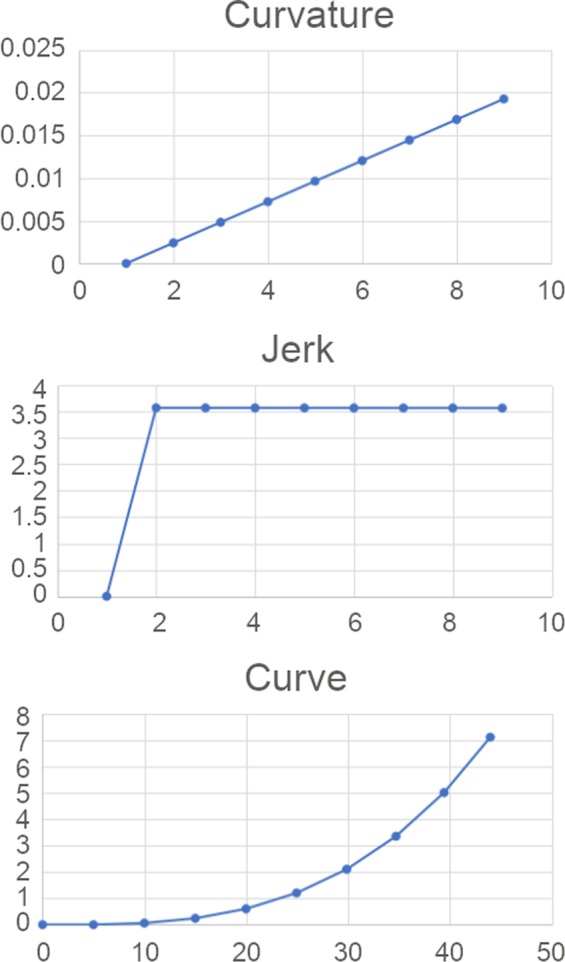
A clothoid curve transition for racing greyhounds with a total 45 m transition length having a an approximately 52 m turning radius at the end of the transition.

Now, for instance, using the numerical method explained above to generate a clothoid curve transition for racing greyhounds with a transition exit radius of approximately 52 m and a total transition length of 45 m, we would have to consider the ***d*** constant to be 0.69 degrees per transition segment, the ***s*** constant to be 5 m (assuming that average stride length of a greyhound is 5 m) and the ***n*** constant to be 9. The curvature and jerk results of this clothoid transition curve for racing greyhounds are shown in Fig. 4. The numerical calculation of ***a***_***i***_ and ***P***_***i***_ is shown in Table 1.

**Table 1:** Numerically calculated values of ***a***_***i***_ and ***P***_***i***_ variables for a clothoid curve.

*i*	a_*i*_ (deg. per segment)	*P*_*i*_ X coordinate (m)	*P*_*i*_ Y coordinate (m)
1	0.69	5.00	0.00
2	2.07	10.00	0.06
3	4.14	15.00	0.24
4	6.9	19.98	0.60
5	10.35	24.95	1.20
6	14.49	29.87	2.10
7	19.32	34.71	3.35
8	24.84	39.43	5.01
9	31.05	43.96	7.11

Using this numerical method approach, we showed how an optimized clothoid curve transition could be determined numerically by tweaking curve generating factors. The controlling of initial values as set by ***d***, ***s***, and ***n*** allows generating any combination of clothoid curves as required for different kinematic path design goals.

### Designing ideal clothoid segments for racing greyhounds and other quadrupeds

When designing clothoid segments, it is essential that greyhound heading is not changing at the maximum performable rate since such a heading would put a greyhound into a limit state turning while maintaining a high speed. An ideal clothoid segment would have continuous curvature to allow a greyhound to navigate the path with a minimal amount of veering effort. In the next section, we derive a helper equation which can be used for specifying ideal clothoid transitions as well as for modelling dynamics for racing greyhounds at the tracks.

### Deriving an equation for exact clothoid requirements for racing greyhounds and other quadrupeds

Equation (3) produced a relationship between greyhound kinematics such as heading turning angle acceleration and turning radius at the end of a natural clothoid transition. First, let’s assume for a clothoid transition a racing greyhound would pass **ns** number of strides with a constant ***s*** meter stride length. Now, if the total clothoid transition length is ***T*** meters, then the number of greyhound strides ***ns*** in a transition is given by Eq. (13). Again, since the length of the greyhound’s strides remains unchanged in the clothoid transition, the greyhound’s turning angle ***a*** in the last stride of the transition can be defined by Eq. (14) if the greyhound heading turning angle is accelerating with ***d*** degrees per stride. Now, to calculate a greyhound’s heading radius of turn ***R*** near the end of the clothoid transition using Eq. (3), we use Heron’s formula (17) to calculate the area of the triangle ***A*** (17) formed by last two greyhound strides ***s***_***1***_ and ***s***_***2***_. Furthermore, using the cosine rule we calculate the unknown side ***s*** of the triangle formed by the last two greyhound strides ***s***_***1***_ and ***s***_***2***_. Finally, by plugging in values for ***R*** and simplifying the equation, we reach a final equation form (18) which defines a racing greyhound’s turning radius ***R*** at the end of the clothoid transition in terms of transition length ***T***, greyhound heading turning acceleration ***a*** and greyhound constant stride length ***s***. Consequently, as Eq. (18) relates greyhound heading turning parameters to clothoid transition parameters, which is useful for modelling and designing ideal clothoid transitions for racing greyhounds. In the next section, we show some of the design and modelling of the clothoid transitions using Eq. (18).

***d*** = transition deflection angle acceleration (per stride)

***a*** = deflection angle of greyhound heading for last greyhound stride

***ns*** = total number of greyhound strides in the transition

***s*** = length of a single stride

***R*** = transition last stride turn radius

***T*** = transition length

13 $$ns=\frac{T}{s}$$

14 $$a=d\times (ns-1)$$

15 $$s=\sqrt{{s}_{1}^{2}+{s}_{2}^{2}-2{s}_{1}{s}_{2}Cos(a-180)}$$

Where ***s***_***1***_ and ***s***_***2***_ are a racing greyhound’s last two strides in the transition.

16 $$p=\frac{{s}_{1}+{s}_{2}+s}{2}$$

Where ***p*** is semi-perimeter of the inscribed triangle (Fig. 1) in the circle formed by a racing greyhound’s last two strides ***s***_***1***_ and ***s***_***2***_.

17 $$A=\sqrt{p(p-{s}_{1})(p-{s}_{2})(p-s)}$$

18 $$R=\frac{\sqrt{2}{s}^{2}\,\sqrt{2{s}^{2}\,\cos \left(\frac{\pi d\left(\frac{T}{s}-1\right)}{180}\right)+2{s}^{2}}}{2\sqrt{-{s}^{4}\left(\cos \left(\frac{\pi d\left(\frac{T}{s}-1\right)}{90}\right)-1\right)}}$$

### Clothoid design for constant radius bend

Every track has a bend radius requirement as calculated from the physical infrastructure and design goals. If a track requires a 52 m radius bend at the end of the transition, then using Eq. (18), we find the following expected greyhound kinematics and transition design possibilities as shown in Table 2. It should be noted that there could be a large number of design outcomes for a single parameter design such as a design for a specific bend radius. The greyhound yaw rate at the entrance is simply the greyhound angular displacement rate change per stride times greyhound stride frequency. Also, in generating the folllowing results racing greyhound speed was assumed to be 19.5 m/s and stride frequency to be 3.5 Hz.

**Table 2:** Clothoid transition options for 52 m radius bend.

Design No.	Clothoid transition length, *T* (m)	*Greyhound yaw rate at the transition entrance (deg/s)	Greyhound angular displacement rate change per stride, *d* (deg/stride^2^)	Greyhound expected constant stride length, *s* (m)
1	75	1.2969	0.393	5.0
2	60	1.6533	0.501	5.0
3	40	2.3825	0.722	4.8
4	60	1.7952	0.544	5.2

As can be seen from Table 2, each of the clothoid transition possibilities can be applied at different locations at the track based on the race requirements. For instance, the clothoid transition Design No. 3 can be applied at the home turn bend exit since the greyhound speed and stride length would be much lower making it possible for a greyhound to adopt to higher yaw rate and angular displacement acceleration path navigation.

### Clothoid design for greyhound angular displacement rate change limits

For racing greyhounds known to have certain angular displacement rate change limits based on greyhound training and health background histories, using Eq. (18) we can enumerate possible clothoid designs options. For example, if the expected racing greyhounds have a maximum angular displacement rate change limit of 0.5 deg/stride^2^, then we can consider the following clothoid transition design options as shown in Table 3.

**Table 3:** Clothoid transition options for racing greyhound accelerating with a maximum angular heading turning of 0.5 degrees per stride^2^.

Design No.	Clothoid transition length, *T* (m)	Radius of constant bent at the transition end (m)	Greyhound expected constant stride length, *s* (m)
1	45	71.6	5.0
2	50	70.5	5.25
3	60	52.0	5.0
4	70	53.7	5.5

As can be seen from Table 3, using greyhound angular displacement rate change as a design constraint exhibits more diverse clothoid transitions in terms of transition length and transition exit bend radius. Design No. 1 shows that it is possible to have a short transition for a larger radius bend. Likewise, design no. 4 portrays a long transition for smaller radius bend. As a result, the angular displacement rate based design approach provides excellent freedom in choosing clothoid transitions based on track requirements.

### Modelling of racing greyhound jerk dynamics

It is possible to calculate jerk exhibited by clothoid transitions using Eq. (18). Since a clothoid has uniform curvature acceleration, the jerk produced by a clothoid remains the same for the entire length of the transition. So, we can find jerk value at any arbitrary location in a clothoid transition to find overall jerk for the transition. For example, if we are interested in the jerk at the end of a clothoid transition, first we would calculate radius value ***R*** for both ***T*** and ***T***-***s*** for the transition. Then, we would calculate corresponding centrifugal acceleration values. Finally, since the jerk is the change in centrifugal acceleration over time, we simply divide the difference of centrifugal accelerations by the time taken by one stride. Table 4 presents some example calculations of racing greyhound jerk values for various clothoid transition designs considering instantaneous greyhound speed to be 19.5 m/s:

**Table 4:** Clothoid transitions racing greyhound’s jerk modelling using Eq. (18).

Design No.	Clothoid transition length, *T* (m)	Radius of constant bend at the transition end (m)	Greyhound expected constant stride length, s (m)	Greyhound angular displacement rate change per stride, *d* (deg/stride^2^)	Absolute jerk (m/s^3^)
1	45	71.6	5.0	0.5	2.59
2	50	70.5	5.3	0.5	2.35
3	60	52.0	5.0	0.5	2.59
4	70	53.7	5.5	0.5	2.14

### An approach to designing ideal transitions for racing greyhounds

As can be seen from Fig. 4, it was found that racing greyhound clothoid transition curves have a significant flaw. Although the development of the curvature is gradual as can be seen from the curvature plot of Fig. 4, the jerk profile is not smooth and almost jumps instantaneously from zero to a higher value (Fig. 4). This is important, as such a dramatic change of jerk would impose a high energy release in a short time resulting in considerably unstable conditions for greyhounds navigating in and out of the transitions. Furthermore, the clothoid curve generation for racing greyhounds using the numerical method above showed that regardless of transition curve length jerk goes through a step change within one transition segment or one racing greyhound stride. Consequently, a clothoid curve transition was deemed not to be an ideal fit for racing greyhound track path designs.

The clothoid transition curve does not maintain a smooth jerk initiation for a racing greyhound. Hence the curve can only be considered G2 continuous with matching curvature at the entrance and exit of the transition curve. This imposes several disadvantages in racing greyhound race dynamics at the tracks. For example, we can break down the disadvantages into two main categories, namely clustering related problems and path smoothing, where each is entangled with the other. The clustering of racing greyhounds is a common issue during races. This happens mainly due to single lure convergence as a result of the number of following galloping greyhounds. A tight convergence of the racing greyhound pack is noticeable at race tracks in the locations where track path curvature change is sudden and abrupt. As clustering is a precursor to various dynamics unstable conditions such as bumping of one greyhound by another, maintaining a smooth path profile such as G3 curvature continuity where the clustering occurs, becomes vital. As greyhounds follow the racing lure, they occupy different lanes such that they have different path radii and tend to cut corners forming various individual transitions into the bend which are all unique. A G2 curvature continuity as found in the clothoid transitions where the rate of change of the jerk is not smooth would induce all the racing greyhounds following the lure to follow one unique transition into the bend to keep instantaneous jerk to the minimum. This is not feasible.

To overcome the limitations of clothoid transitions, we applied the numerical method of generating clothoid curves discussed in the previous section to develop moderate G3 curvature continuity transition curves for racing greyhounds. Also, two different transition curve configurations were selected for generating the curves as these configurations best match the many current tracks found in Australia in terms of real estate requirements. The configurations are a 45 m transition with transition end radius of 52 m and a 75 m transition with transition end radius of 70 m.

**Figure 5:**
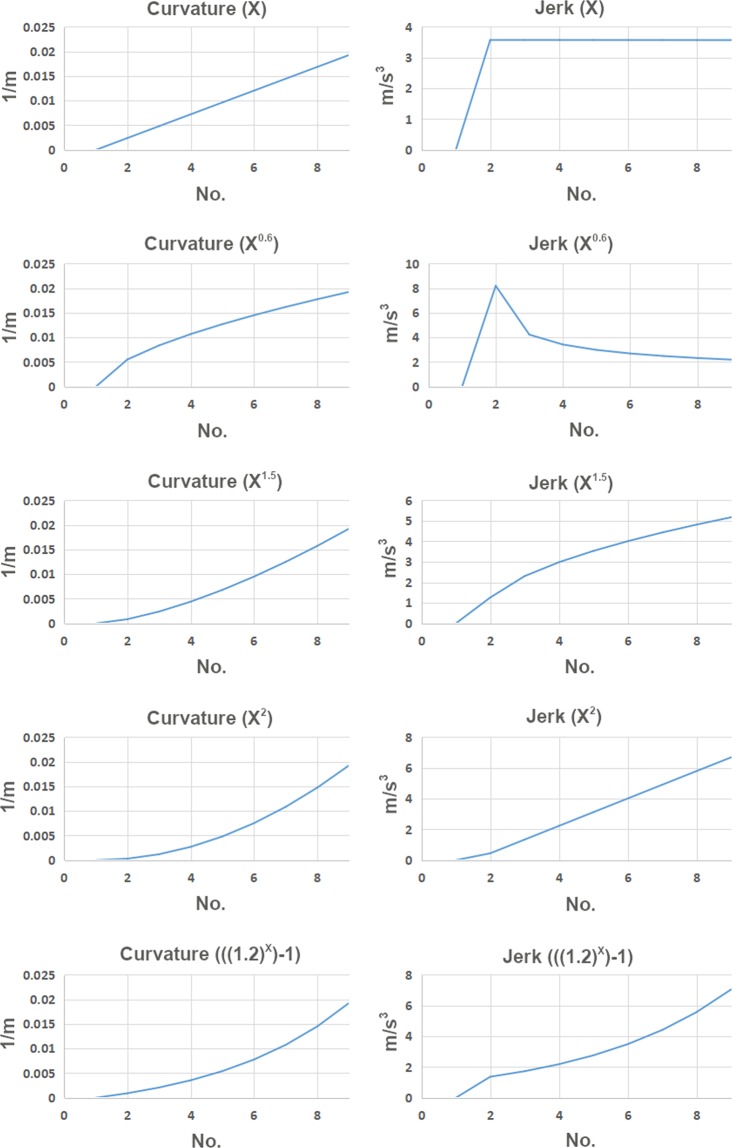
Different smooth curves curvature and jerk results as 45 m transition curves for greyhound racing.

**Figure 6:**
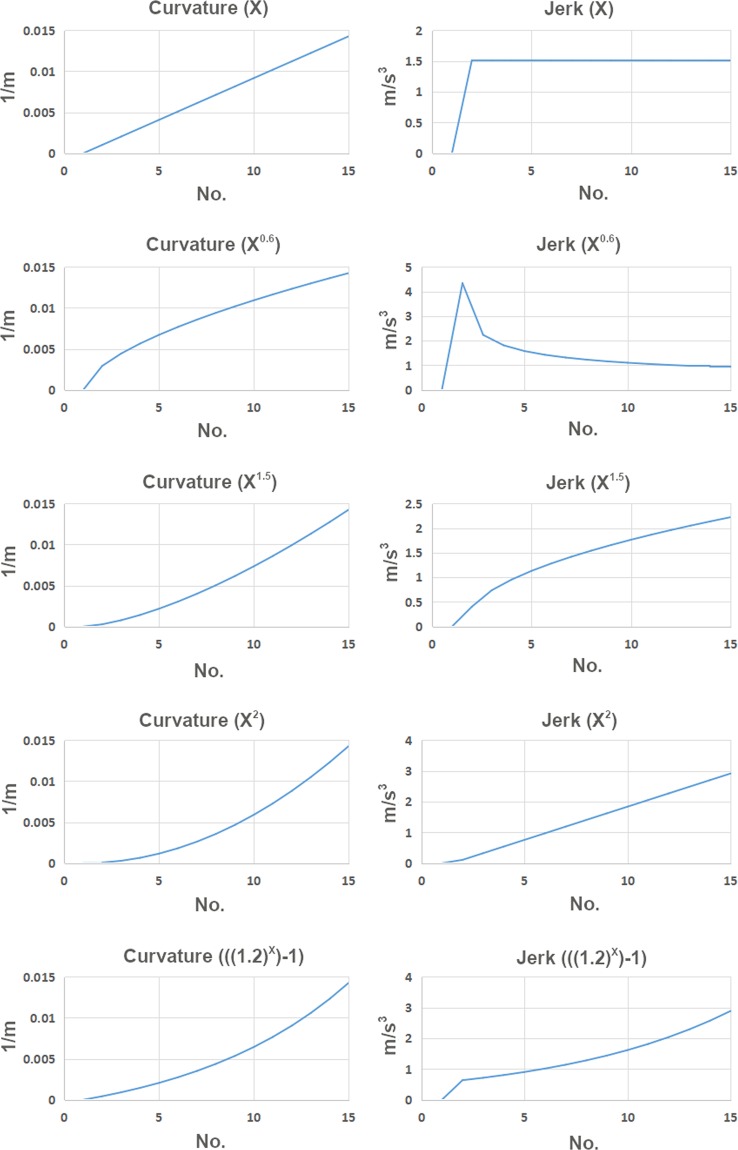
Different smooth curves curvature and jerk results as 75 m transition curves for greyhound racing.

First, we assume ***a***_***i***_ = ***X*** and plot for different ***X*** expressions to derive different curves where the curvature results for the curves are shown in Figs. 5 and 6. The ***X*** expression defines the nature of curvature function as the curve length increases from the origin. As seen from the plots (Figs. 5 and 6), when the ***X*** expression is linear it is a clothoid transition where the jerk is initiated immediately within one transition segment for both 45 m and 75 m transition configurations. To get G3 curvature continuity curves, we tried ***X***^***0.6***^, ***X***^***1.5***^, ***X***^***2***^, and ***((1.2)***^***X***^***−1)*** expressions. As can be seen from the plots, all the curves except the clothoid curve ***X*** and ***X***^***0.6***^ curve maintain a moderate G3 curvature continuity with a smooth jerk profile. However, as ***X*** expressions are in power and logarithmic function form for ***X***^***0.6***^, ***X***^***1.5***^, ***X***^***2***^, and ***((1.2)***^***X***^***−1)*** these curves result in higher jerk in the second half of the transition. This suggested that ***X***^***1.5***^, ***X***^***2***^, and ***((1.2)***^***X***^***−1)*** curves could be used to develop a G3 curvature continuity transition curve for racing greyhounds if the jerk could be maintained in the second half of the transition. Thus, we decided to use these curves as auxiliary curves which would provide smooth jerk initiation for the transition. However, compared to other curves, the overall jerk and smoothness performance of the ***X***^***1.5***^ is optimum.

Here, we generate composite transition curves with various degrees of G3 curvature continuity for racing greyhound ideal path design. Each composite transition curve generated combines the ***X***^***1.5***^ curve as an auxiliary curve and a clothoid curve as the main curve. So, the overall transition curve generating function can be considered as a piecewise function shown in Eq. (19) where the auxiliary curve function g is applicable until ***q*** transition segment is reached.

19 $$f(x)=\{\begin{array}{c}g(x)\\ z(x)\end{array}\begin{array}{c}{\rm{if}}\,x < q\\ {\rm{otherwise}}\end{array}$$

Where,

$$x=y(d,i)$$

**Figure 7:**
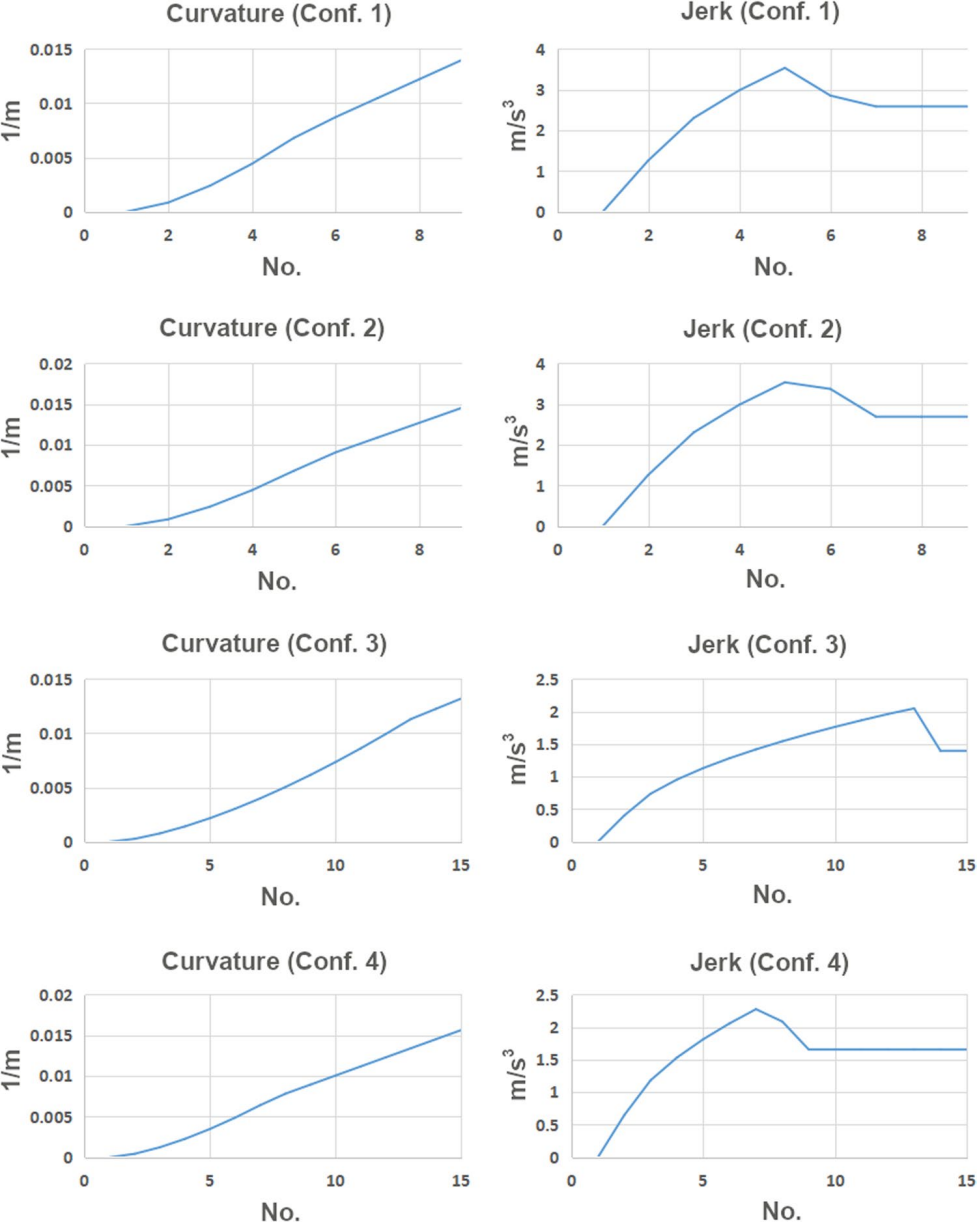
Four different straight to bend curvature graphs and jerk results for ideal racing greyhound transition curves.

Figure 7 shows curvature and jerk results for four different composite curves as ideal transitions for racing greyhounds, plotted using the numerical method explained in the earlier section, the configurations for these composite curves are given in Table 5.

**Table 5:** Kinematic and shape properties for four straight to bend composite curve transitions.

Composite curve configuration No.	Transition deflection angle acceleration for auxiliary curve (deg.)	Transition deflection angle acceleration for main clothoid curve (deg.)	Total transition length (m)	Transition exit radius (m)
1	0.3900	0.50	45	71.6
2	0.3900	0.52	45	68.9
3	0.1825	0.27	75	75.8
4	0.2500	0.32	75	64.0

As can be seen from Fig. 7, composite transition curves have strong advantages over pure clothoid transition in terms of curvature and jerk continuities and excellent moderate G3 continuity for the first half of the transition. The overall instantaneous jerk is significantly lower in the composite curve transitions compared to clothoid transitions. This is because the window of jerk initiation is much longer in composite curve transitions because of the gradual development of jerk and on average it is four transition segments or four greyhound strides compared to just one stride in the clothoid transitions.

## Greyhound racing data results

A racing greyhound getting injured at the tracks provides an indication of its overall racing trajectory performance. Also, we can analyze the trajectory of a racing greyhound at the tracks to measure track path performance. Below, we present two such case scenarios by analyzing racing greyhound track data and injury rates.

### Race injury data results for track path renovation

In the greyhound racing track path design, it was found that only circular arcs (constant curvature) and lines (zero curvature) were used extensively despite non-continuous curvature resulting at the segment intersections^25^. A discontinuity in the curvature implies that a greyhound must change its heading instantaneously and abruptly resulting in a path which is not feasible^8^. Also, track survey data from Australia shows that a brief transition is applied, made of an arc spline consisting of one or more circular arcs joined with continuous tangent vectors. This particular design practice also leads to multiple discontinuities in track path curvature.

**Figure 8:**
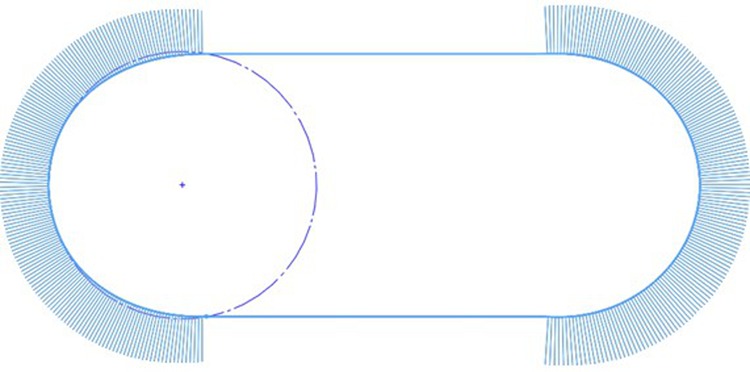
Track path curvature as shown by curvature combs for Track A with G1 curvature continuity for bends.

**Figure 9:**
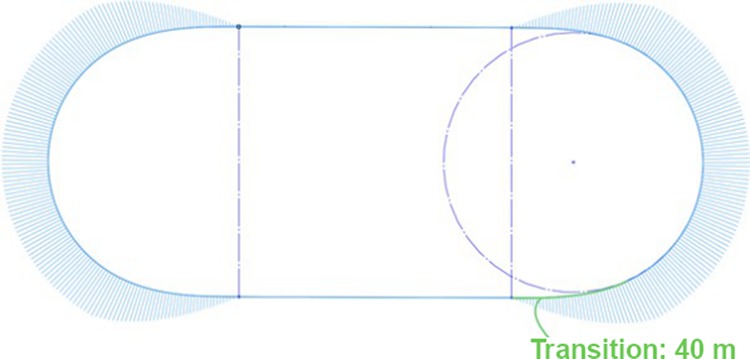
Track path curvature as shown by curvature combs for Track A with G2 curvature continuity for bends.

We looked into one particular greyhound racing track (Track A) located in Australia and its two years of racing history. In the first year, it had a track path design with G1 continuity constituting half-circle bends and straights (Fig. 8). In the second racing year, the track was renovated with clothoid curve transitions into and out of the constant radius bends (Fig. 9). A 40 m clothoid transition was adjoined between a straight and a constant bend section for four bend and straight intersections. The outcome of this clothoid transition incorporation into the original track path design eases centrifugal acceleration effect on the greyhounds where the centrifugal force is raised gradually from zero to an approximate nominal 240 N (Fig. 9). The renovation at Track A definitely would have changed centrifugal jerk performance significantly as the clothoid curve joining straights and bends would maintain G2 curvature continuity for the track path. To see whether this resulted in a significant decrease in racing injury rates, injury data for a two-year period were analyzed containing one year injury data for before and after renovation. By assuming differences in other contributing factors to injury rates such as variations in weather, track maintaining conditions, different greyhound breeds and training patterns, race operating conditions between the years were minimal the injury rates should show general trends due to track path renovation changes. We found that before the clothoid intervention at Track A the normalized catastrophic and major injury rate per 1000 race starts was about 4.58 whereas after the clothoid intervention it was reduced to 4.22, a 7.9% reduction in this category of injury rates. Typically, this category of injury results from significant damage to greyhound physics. However, when we took into account all types of injuries at Track A for before and after the renovation, the normalized injury rates per 1000 race starts reduced to 26.71 from 44.68 injuries, a 40.2% reduction in overall injuries due to clothoid implementation at the track. Furthermore, under all injury types the most commonly occurring injury is happening in the greyhound forelegs responsible for turning assist for dog’s navigation. Metacarpal fractures and tibial fractures due to torsional stress occurring in the forelegs indicate navigational work stress on the greyhounds.

### Curvature of racing greyhound trajectory

**Figure 10:**
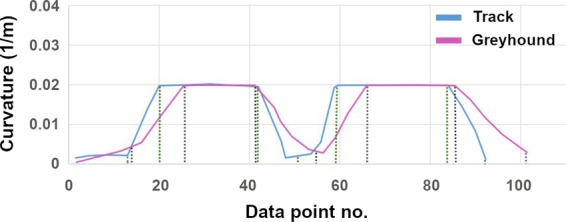
Track path and greyhounds trajectory curvature comparison.

Like any path following object, a racing greyhound has limitations of the radius of curvature or extrema of curvature for its running path. Also, when a racing greyhound runs following a track path which has curvature discontinuity or non-optimal transitions, a deviation in the greyhound’s position occurs from the projected track path trajectory. This phenomenon was observed in the greyhound location data in the races. Furthermore, numerical racing greyhound simulations confirmed that when a greyhound is following the line of sight of a lure, its yaw rate gradually builds up for the bend for a track shape which is less circular^5^. To see if there is any difference between racing greyhound path trajectory to track path, we analyzed racing greyhound location tracking data for a track which has non-optimal transition length to reduce the jerk magnitude. From the racing greyhound location tracking data and track survey data, we generated curvature results for both racing greyhound trajectory and track path (Fig. 10). The greyhound location data for all greyhounds starts were averaged to plot the results where ten races or eighty starts were considered to plot the results below. As can be seen from the curvature plot, there is a significant difference between racing greyhound trajectory and track path. This indicates racing greyhounds deviating from the track path to accommodate a more natural trajectory according to their physics. Also, it was observed from the analysis that transitions occurring in racing greyhound trajectory is relatively gradual and longer as indicated by the green dashed marker compared to the black dashed marker for track path.

## Conclusions

This paper presents a numerical method for generating racing greyhound clothoid transitions for track path designs along with an equation for modelling any kind of clothoid curves. The numerical technique is robust and can be algorithmically controlled to achieve defined goals compared to existing approaches for designing racing greyhound clothoid transitions. Moreover, it can be extended to function as a generator of other curves rather than just clothoid curves. By looking into jerk modelling data, an ideal transition curve is presented suitable for racing greyhound track path designs which overcomes limitations set by clothoid transitions. The effect of clothoid transitions in an existing track was verified by measuring injury rates over a two-year period. The trajectory of racing greyhounds in an existing track with inadequate transitions was analyzed to show non-optimum track path conditions.

Finally, this paper showed evidence through modelling and injury data that clothoid and other composite curves improve racing dynamics safety for racing greyhounds. Furthermore, the methods presented here can also be used in designing and modelling trajectories for other moving bodies, including but not limited to horses, vehicles and trains.
